# Unripe *Rubus coreanus* Miquel Extract Containing Ellagic Acid Regulates AMPK, SREBP-2, HMGCR, and INSIG-1 Signaling and Cholesterol Metabolism In Vitro and In Vivo

**DOI:** 10.3390/nu12030610

**Published:** 2020-02-26

**Authors:** Ki Hoon Lee, Eui-Seon Jeong, Goeun Jang, Ju-Ryun Na, Soyi Park, Wan Seok Kang, Eun Kim, Hakjoon Choi, Jin Seok Kim, Sunoh Kim

**Affiliations:** B&Tech Co., Ltd., Central R&D Center, Gwangju 61239, Korea; leekh7@epost.kr (K.H.L.); 22c_goldskin@naver.com (E.-S.J.); goeun2748@gmail.com (G.J.); ryun1225@daum.net (J.-R.N.); soyipark@hanmail.net (S.P.); kws2602@hanmail.net (W.S.K.); rubsang84@gmail.com (E.K.); ohchj12@naver.com (H.C.); keki2000@naver.com (J.S.K.)

**Keywords:** unripe *Rubus coreanus*, cholesterol, hypercholesterolemia, AMPK, SREBP-2, HMGCR

## Abstract

Our previous study demonstrated that a 5% ethanol extract of unripe *Rubus coreanus* (5-*u*RCK) has hypo-cholesterolemic and anti-obesity activity. However, the molecular mechanisms of its effects are poorly characterized. We hypothesized that 5-*u*RCK and one of its major bioactive compounds, ellagic acid, decrease cellular and plasma cholesterol levels. Thus, we investigated the hypocholesterolemic activity and mechanism of 5-*u*RCK in both hepatocytes and a high-cholesterol diet (HCD)-induced rat model. Cholesterol in the liver and serum was significantly reduced by 5-*u*RCK and ellagic acid. The hepatic activities of HMG-CoA and CETP were reduced, and the hepatic activity of LCAT was increased by both 5-*u*RCK extract and ellagic acid, which also caused histological improvements. The MDA content in the aorta and serum was significantly decreased after oral administration of 5-*u*RCK or ellagic acid. Further immunoblotting analysis showed that AMPK phosphorylation in the liver was induced by 5-*u*RCK and ellagic acid, which activated AMPK, inhibiting the activity of HMGCR by inhibitory phosphorylation. In contrast, 5-*u*RCK and ellagic acid suppressed the nuclear translocation and activation of SREBP-2, which is a key transcription factor in cholesterol biosynthesis. In conclusion, our results suggest that 5-*u*RCK and its bioactive compound, ellagic acid, are useful alternative therapeutic agents to regulate blood cholesterol.

## 1. Introduction

Hypercholesterolemia is the most important risk factor for cardiovascular disease (CVD). Thus, aggressive diagnosis and management to lower excessive detrimental lipids are necessary [1,2]. Statins, which are 3-hydroxy-3-methyl-glutaryl-CoA reductase (HMGCR) inhibitors, are strongly recommended for clinical treatment because of their characteristics including their high level of tolerance, various protective effects on the cardiovascular system, and ability to lower plasma low-density lipoprotein cholesterol (LDL-C) [3,4]. However, some complications of statins have been reported such as muscle-related symptoms [5,6] and depression [7]. It was also reported that statins do not protect against cardiovascular events in patients who are above 70 years of age [8]. Therefore, the use of natural substances has been suggested as an alternative strategy for the reduction of LDL-C levels.

An understanding of the molecular basis of anti-hypercholesterolemic effects is necessary to assess the effects of natural substances. One potential target for dietary intervention against metabolic disorders is AMP-activated protein kinase (AMPK), which is a cellular sensor of energy metabolism and a regulator of cholesterol and glucose metabolism and hepatic lipid metabolism [9,10,11]. AMPK directly phosphorylates sterol regulatory element binding protein-2 (SREBP-2), which inhibits the nuclear translocation of SREBP-2 and downregulates HMGCR transcription [12]. Furthermore, activation of insulin-induced gene 1 (INSIG-1), which targets AMPK by drugs, leads to reduced levels of active SREBP and, consequently, to reduced target gene expression including the genes responsible for triglyceride (TG) synthesis [13]. Collectively, these results demonstrate that the activation of AMPK and suppression of SREBP-2 may lead to reduced LDL-C levels.

The *Rubus* genus comprises thousands of species of blackberries and raspberries grown worldwide, and species in this genus have been investigated as novel therapeutic agents for a metabolic syndrome through their beneficial effects [14]. One such species, *Rubus coreanus* Miquel, which is native to Eastern Asia, has been used as a traditional alternative medicine to manage impotence, spermatorrhea, enuresis, asthma, and allergic diseases [15]. In fact, the small fruits of this species contain many nutrients such as minerals, vitamins, and sugars as well as phenolic compounds including phenolic acids, flavonoids, and tannins [16]. Thus, the many beneficial effects of *R. coreanus* Miquel, such as its antitumor, antioxidative, anti-inflammatory, and anti-obesity effects have been reported [17,18]. One of the major active compounds in *R. coreanus* is ellagic acid, which has anti-obesity and antioxidant properties [19,20]. The unripe fruits of *R. coreanus* Miquel are usually used as traditional medicine and might be more therapeutic than the ripe fruits. A recent report revealed that the unripe fruits of *R. coreanus* have a higher concentration of ellagic acid than the ripe fruits, which might be the reason why unripe fruits are traditionally used [21]. Our previous study demonstrated the anti-obesity [22,23] and anti-hypercholesterolemic effects [24] of an extract of unripe *R. coreanus* (5-*u*RCK) cultivated in Korea in a high-fat diet (HFD)-fed and high-cholesterol diet (HCD)-fed mice, and we suggested that five bioactive compounds contribute to the decreased lipid accumulation in 3T3-L1 adipocytes demonstrated in another report [23]. We studied the effect of 5-*u*RCK and various species of *Rubus* through in vitro assays to identify candidates for treatment that were more effective than 5-*u*RCK. Among the candidates, a 5% ethanol extract of unripe *R. chingii* (5-*u*RCC) cultivated in China showed the highest effective for reducing cholesterol accumulation, except for those of 5-*u*RCK, in vitro in our previous screening study (unpublished data). Furthermore, our previous study reported that ellagic acid is a major component of 5-*u*RCC [25]. Thus, our previous studies in animal models demonstrated that 5-*u*RCK exerts an anti-hypercholesterolemic effect. However, its effect on cholesterol metabolism is unknown. Furthermore, the hypercholesterolemic effect of 5-*u*RCC has not yet been explored. The purpose of this study is to compare anti-hypercholesterolemic effect of 5-*u*RCK with 5-*u*RCC, and to investigate the possibility of mixing or substitution as raw materials with anti-hypercholesterolemic effects.

Therefore, the present study was designed to compare the effects of 5-*u*RCK and 5-*u*RCC extracts on cholesterol metabolism in human HepG2 cells. We also investigated the antioxidative status and molecular mechanisms of action of 5-*u*RCK in rats fed a hypercholesterolemic diet.

## 2. Materials and Methods

### 2.1. Reagents

Simvastatin, oleate, palmitate, and ellagic acid were purchased from Sigma-Aldrich (St. Louis, MO, USA). Dulbecco’s modified Eagle’s medium (DMEM), bovine serum albumin (BSA), and fetal bovine serum (FBS) were purchased from Invitrogen, Inc. (Grand Island, NY, USA). All other chemicals were of an analytical reagent grade.

### 2.2. Preparation of Extracts

The unripe *R. coreanus* (*u*RCK, specimen voucher number: BT-URCK001) fruits used in this study were collected (May 2017) in Gochang County (Jeollabuk-do, Korea) and authenticated by Dr. Kim at B&Tech, Gwangju, South Korea. The unripe *R. chingii* (*u*RCC, specimen voucher number: BT-URCC001) fruits used in this study were obtained from the Seoul pharmaceutical market (South Korea) and authenticated by Dr. Kim at B&Tech. *u*RCK or *u*RCC (2.5 kg) were extracted using 20 volumes of 5% ethanol at 100 °C for 4 h. The extracted solution was then filtered, concentrated with an evaporator under a vacuum, and freeze-dried. The dry matter content of the lyophilized samples was determined by drying at 105 °C to a constant mass (Table 1).

### 2.3. Fractionation and HPLC Analysis of Extracts

The dried 5-*u*RCK or 5-*u*RCC (30 g) was suspended in distilled water and successively divided with *n*-hexane (3 × 500 mL), chloroform (CHCl_3_, 3 × 500 mL), ethyl acetate (EtOAc, 3 × 500 mL), and *n*-butanol (BuOH, 3 × 500 mL). After the final extracts were collected, the amounts of ellagic acid in the extracts were analyzed with high-performance liquid chromatography (HPLC) and compared with a standard preparation of ellagic acid produced with our previously reported standard method [25].

### 2.4. Isolation of Ellagic Acid (EA)

The ethyl acetate fraction of 5-*u*RCK (18 g) was loaded onto a silica gel column (length: 32 cm, diameter: 2 cm, Merck, Darmstadt, Germany) and eluted with a stepwise gradient of chloroform, ethyl acetate, and methanol. The collected active subfractions were further separated by preparative thin layer chromatography (TLC) since the active isolate was not pure. It was again purified on an LH-20 column (length: 20 cm, diameter: 1.5 cm) using methanol as the eluant. The isolated compound was pure by HPLC.

### 2.5. Cell Culture and Cellular Total Cholesterol, Free Cholesterol, and Cholesterol Ester Content Measurements

The HepG2 cell line (88065) was obtained from the Korea Cell Line Bank (KCLB, Seoul, Korea). Each extract was dissolved in culture media, sterilized by filtration with sterile filters (0.2 µm), and diluted to the required concentration. After incubation with 5-*u*RCK and 5-*u*RCC at the indicated concentration for 24 h, the total cholesterol (TC), free cholesterol (FC), and cholesterol ester (CE) content in HepG2 cells was determined using a cholesterol/cholesteryl ester kit (Abcam, Cambridge, MA, USA). Spectrofluorimetric measurements were performed using a spectrofluorimeter (535-nm excitation/587-nm emission wavelengths, Tecan, Männedorf, Switzerland), and the values obtained were normalized to the total protein content, as measured by the Lowry method.

### 2.6. Induction of Fat Overload and Intracellular Total Cholesterol Content Measurement in HepG2 Cells

To induce cellular fat overload, HepG2 cells at 75% confluency were exposed to a mixture of long-chain free fatty acids (FFAs) (oleic acid and palmitic acid) at a 2:1 ratio. After reaching 75% confluence, the HepG2 cells were serum-starved for 18 h and exposed to 1 mM FFA, with or without 5-*u*RCK, 5-*u*RCC, 5-*u*RCK fractions, and ellagic acid for 24 h. The TC content in the HepG2 cells was determined using a total cholesterol assay kit (Abcam, Cambridge, MA, USA). The Lowry method estimated protein content and was used to calculate the amount of cholesterol.

### 2.7. Animal and Experimental Groups

Four-week-old male Sprague-Dawley (SD) rats weighing 100–120 g were purchased from Central Lab Animal, Inc. (Seoul, Republic of Korea). All the experimental procedures were conducted in accordance with the relevant guidelines for the care of experimental animals and approved by B&Tech (approval number BT-006-2017). Rats were fed a normal-cholesterol diet (NCD, AIN-93G, Research Diets, D10012G, New Brunswick NJ, USA, Table 2) or an HCD (Paigen’s atherogenic rodent diet, Research Diets, D12336, New Brunswick, NJ, USA, Table 2) for 3 weeks. Then, the animals were randomly assigned to the following eight treatment groups consisting of 8 rats each (*n* = 8 per group, Table 3). This dosage was calculated from an amount of 600 mg of 5-*u*RCK extract per day per 60 kg of human body weight. All extracts were dissolved in physiological saline solution and the treatments were administered orally by gavage using a stainless oral zonde (Jungdo-BNP, Seoul, South Korea) and the control rats received the same volume of saline solution. Food and water intake was measured every day, and body weight was measured once every two days. The feed efficiency ratio (FER) was calculated as follows: FER = *G* × *F*^−1^ where *G* is the weight gain (g) and *F* is the consumption (g) of dry matter from the feed [22,23,24,26]. At 8 weeks of feeding, rats were anesthetized with isoflurane, and blood samples were collected by cardiac puncture. The liver, kidney, and spleen were snap-frozen in liquid nitrogen or fixed in 4% formaldehyde for histological analysis. During the longer-term treatment, a period from 3 weeks to 8 weeks, death (not drug related) occurred in the NCD group (1 rat), the EA 2 group (2 rats), and the EA 4 group (1 rat) due to the insertion of drugs into the bronchus. There were no deaths in the other groups during the treatment period (Table 3).

### 2.8. Analysis of Biochemical Parameters

The concentrations of serum and hepatic TC, triglyceride (TG), high-density lipoprotein-cholesterol (HDL-C), and low-density lipoprotein/very-low-density lipoprotein cholesterol (LDL/VLDL-C) were measured using an Alere cholesterol LDX^®^ system (Cholestech LDX, Hayward, CA, USA). Aspartate aminotransferase (AST) and alanine aminotransferase (ALT) assays were performed with a kit according to the manufacturer’s instructions (Asan Pharm, Seoul, Korea). Apolipoprotein A1 (Apo A1) and apolipoprotein B (Apo B) assays were performed with a kit according to the manufacturer’s instructions (Novus Biologicals, Littleton, USA).

The atherogenic index (AI_1_) was calculated [27] as follows:

AI_1_ = (TC-HDL-cholesterol)/HDL-cholesterol (1)

The cardiac risk index (CRI) was calculated [26] as follows:

CRI = TC/HDL-cholesterol(2)

### 2.9. Liver Lipid Content Analysis

The liver lipids were extracted as described by Folch et al. [28], and the total cholesterol concentration was measured using the corresponding kits (DRI-CHEM 4000i, Fujifilm, Tokyo, Japan) in accordance with the manufacturer’s instructions. The TC, FC, CE, FC/CE ratio, and protein were calculated, according to the methods reported by Lee et al. [29].

CE levels were calculated by subtracting FC levels from TC levels.

FC/CE ratio levels were calculated as follows:

FC/CE ratio = FC/(TC − FC)(3)

After the lipids were extracted by organic solvent, the remainders were lysed in cell lysis buffer and then the protein concentration was determined by the Lowry method.

### 2.10. Liver Antioxidant Enzyme Analysis

The total antioxidant capability (TAOC), the superoxide dismutase (SOD), glutathione peroxidase (GSH-Px) and catalase (CAT) antioxidant activities, and the malondialdehyde (MDA) and protein carbonyl content in liver homogenates were determined with enzymatic methods using corresponding commercial kits (Cayman Chemical Company, Ann Arbor, MI, USA).

### 2.11. Histological Analysis of the Liver and Aorta

The livers and aortas were fixed in 4% formaldehyde and stored at −80 °C. Five-micron-thick sections of the frozen liver and aortic tissues were prepared using a cryomicrotome and stained with hematoxylin and eosin (H&E). The images were acquired by light microscopy (Olympus BX51, Tokyo, Japan), and we analyzed the images with MetaMorph^®^ microscopy automation and image analysis software (Molecular Devices, Sunnyvale, CA, USA).

### 2.12. Hepatic Cholesteryl Ester Transfer Protein (CETP), Lecithin Cholesterol Acyltransferase (LCAT), and 3-Hydroxy-3-Methylglutary CoA (HMG-CoA) Reductase Activity

The cholesteryl ester transfer protein (CETP) activity in the liver was measured using a CETP inhibitor drug screening kit (BioVision Co., Ltd., Palo Alto, CA, USA), according to the manufacturer’s instructions. The LCAT activity in the liver was measured using a Lecithin cholesterol acyltransferase activity assay kit (Cell Biolabs, Inc., San Diego, CA, USA), according to the manufacturer’s instructions. HMG-CoA reductase enzyme activity in liver homogenates was assayed indirectly by employing the method of Rao and Ramakrishnan [30].

### 2.13. Determination of Thiobarbituric Acid-Reactive Substances (TBARS)

Lipid peroxidation in the blood, liver, and aorta was measured using a TBARS assay kit (R&D Systems, Minneapolis, MN, USA), according to the manufacturer’s instructions. The TBARS concentrations were calculated using a standard curve for 1,1,3,3-tetraethoxypropane (TEP) (0–16.7 μmol/L) and are expressed as the percentage of MDA production.

### 2.14. RNA Extraction and Reverse Transcription-Polymerase Chain Reaction (RT-PCR)

Total RNA was extracted from the liver tissue using an easy-BLUE total RNA extraction kit (iNtRON Biotechnology, Seongnam, Republic of Korea), according to the manufacturer’s instructions. To synthesize cDNA, 1 ug of total RNA was mixed with a premixture of oligo (dT) primer and incubated at 45 °C for 60 min. The specific primers that we used in this study were as follows: SREBP-2 sense 5′-ATCCGCCCACACTCACGCTCCTC-3′, antisense 5′-GGCCGCATCCCTCGCACTG-3′, HMGCR sense 5′-AAGGGGCGTGCAAAGACAATC-3′, antisense 5′-ACACGGCACGGAAAGAACCATAGT-3′. The sense (5′-GGCACAGTCAAGGCTGAGAATG-3′) and antisense (5′-ATGGTGGTGAAGACG CCAGTA-3′) primers for glyceraldehyde 3-phosphate dehydrogenase (GAPDH) were used as a control to measure the total RNA content of each sample. Expression levels were quantified using a gel documentation and analysis system (ChemiDoc XRS+ System, Bio-Rad, Sydney, Australia). The relative expression levels of target genes were normalized to a GAPDH internal control.

### 2.15. Protein Extraction and Immunoblot Assays

Samples of liver tissue were washed three times with cold phosphate-buffered saline (PBS) before being lysed in radioimmunoprecipitation (RIPA) lysis buffer (10 mmol/L Tris-HCl, pH 7.5, 1% NP-40; 0.1% sodium deoxycholate, 0.1% SDS, 150 mmol/L NaCl, and 1 mmol/L EDTA) supplemented with 1× protease and phosphatase inhibitor cocktail (Thermo, Fremont, CA, USA) on ice. The separated proteins were transferred onto a nitrocellulose membrane. The monoclonal anti-AMPK (1:100), anti-phospho-AMPK (1:200, Thr172), anti-HMGCR (1:100), anti-phospho-HMGCR (1:100, Ser872), anti-SREBP-2 (1:100), INSIG-1 (1:100), and anti-β-actin (1:3000) antibodies and secondary antibodies (1:10,000) were obtained from Abcam (Cambridge, MA, USA). Immunoreactivity protein bands were visualized using a ChemiDoc XRS+ System (Bio-Rad), and quantified with Gel Pro Analyzer software (Silk Scientific, Inc., Orem, UT, USA). The internal control, β-actin, was used to normalize differences due to loading variations.

### 2.16. Statistical Analysis

Data are presented as the mean standard deviation (SD) or standard error (SE) from three independent experiments with replication. Data were statistically evaluated using Student’s *t*-test or one-way analysis of variance (ANOVA) with GraphPad Prism 5 (GraphPad, Inc., San Diego, California, USA) software programs. Differences between groups were assessed using Duncan’s multiple range tests. Statistical significance was indicated when *p* < 0.05.

## 3. Results

### 3.1. 5-uRCK and 5-uRCC Reduce Intracellular Cholesterol Concentrations in Hepatocytes

**Figure 1:**
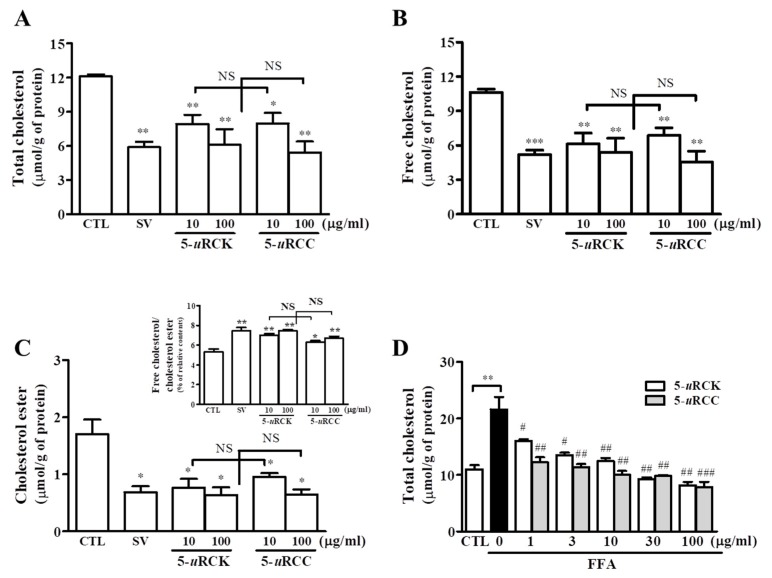
Effects of 5-*u*RCK and 5-*u*RCC on hepatic cholesterol accumulation. HepG2 cells were treated with 5-*u*RCK, 5-*u*RCC, or simvastatin (SV) for 24 h. The cellular total cholesterol (**A**), free cholesterol (**B**), and cholesterol ester (**C**) levels and free cholesterol-to-cholesterol ester ratio (C insert) under normal culture conditions were quantified. (**D**) HepG2 cells were treated with a 1 mM FFA mixture (oleic acid/palmitic acid, 2:1) and various concentrations of 5-*u*RCK or 5-*u*RCC (0–100 μg/mL) for 24 h. * *p* < 0.05, ** *p* < 0.01, *** *p* < 0.001 versus control (CTL). ^#^ *p* < 0.05, ^##^ *p* < 0.01, ^###^ *p* < 0.001 versus FFA-treated cells. NS: not significant. The data are represented as the means ± SEMs.

Our previous in vitro and in vivo experimental results suggested that 5-*u*RCK downregulated lipid and cholesterol accumulation to the greatest extent among the extracts tested [22,23,24]. Therefore, we screened for an optimal condition to reduce cholesterol levels using highly similar species of the *Rubus* genus: 5-*u*RCK and 5-*u*RCC. The cytotoxicity of 5-*u*RCK or 5-*u*RCC was evaluated by an 3-(4,5-dimethylthiazol-2-yl)-2,5-diphenyltetrazolium bromide (MTT) assay. The viability of cells treated with different concentrations (1 μg/mL, 3 μg/mL, 10 μg/mL, 30 μg/mL, and 100 μg/mL) of 5-*u*RCK or 5-*u*RCC compared to control cells was 98.76%, 98.74%, 95.88%, 96.29%, and 94.91% (Figure S1). 5-*u*RCK or 5-*u*RCC did not affect MTT activity, which suggests that 5-*u*RCK or 5-*u*RCC had no cytotoxic effect under the present experimental conditions. As shown in Figure 1, 5-*u*RCK and 5-*u*RCC significantly reduced the cellular TC, FC, and CE concentrations after 24 h of culture, and the decrease in TC with 5-*u*RCK or 5-*u*RCC treatment was apparently dose-dependent. Intracellular cholesterol measurements revealed that 5-*u*RCK (10 μg/mL or 100 μg/mL) significantly reduced TC, FC, and CE concentrations by 34.50–49.55%, 42.53–49.24%, and 55.26–62.71%, respectively, compared with those in controls. The ratio of FC to CE was also significantly increased in 5-*u*RCK-treated cells (5.31 vs. 7.44 for control and 5-*u*RCK at 100 μg/mL, respectively, *p* < 0.01) (Figure 1C, insert). All 5-*u*RCC extracts downregulated TC, FC, and CE concentrations in a dose-dependent manner. However, the effects of 5-*u*RCK and 5-*u*RCC were not significantly different (*p* > 0.05). As shown in Figure 1D, TC quantification revealed that 5-*u*RCK and 5-*u*RCC at treatment concentrations of 0–100 μg/mL dose-dependently attenuated FFA-induced TC accumulation compared to that in the group treated with FFA alone.

### 3.2. Identification of Ellagic Acid in Fractions of 5-uRCK and 5-uRCC Using HPLC Analysis

**Figure 2:**
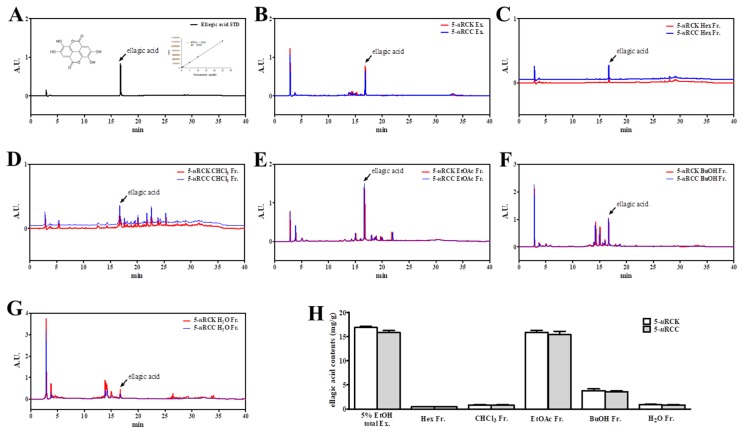
HPLC-diode array detector (HPLC-DAD) chromatograms of the major components of 5-*u*RCK or 5-*u*RCC and their fractions. The peak with a retention time of 16.5 min was assigned to ellagic acid. Ellagic acid was identified at a wavelength of 254 nm. Chromatograms of standard ellagic acid (**A**), 5% ethanol extracts of *u*RCK or *u*RCC (**B**), *n*-hexane fractions of 5-*u*RCK or 5-*u*RCC (**C**), chloroform fractions of 5-*u*RCK or 5-*u*RCC (**D**), ethyl acetate fractions of 5-*u*RCK or 5-*uRCC* (**E**), *n*-butanol fractions of 5-*u*RCK or 5-*u*RCC (**F**), and water fractions of 5-*u*RCK or 5-*u*RCC (**G**). (**H**) The amount of ellagic acid in the extracts and fractions of 5-*u*RCK or 5-*u*RCC. The data are represented as the means ± SDs.

The yields of 5-*u*RCK and 5-*u*RCC following 5% ethanolic extraction were 20.44% and 19.54%, respectively (Table 1). Figure 2 shows the chromatograms of 5-*u*RCK, 5-*u*RCC, and their fractions from HPLC analysis. The chromatographic profiles of 5-*u*RCK and 5-*u*RCC showed the presence of one major compound, which was identified as ellagic acid. Its concentration in 5-*u*RCK and 5-*u*RCC was 16.84 and 15.79 mg/g, respectively. After fractionation, ellagic acid was present in all fractions. However, the EtOAc fraction gave the richest ellagic acid profile. The ellagic acid content of five fractions of 5-*u*RCK was 0.44 mg/g (Hexane Fr.), 0.82 mg/g (CHCl_3_ Fr.), 15.84 mg/g (EtOAc Fr.), 3.82 mg/g (BuOH Fr.), and 0.92 mg/g (H_2_O Fr.). Additionally, the ellagic acid content of five fractions of 5-*u*RCC was 0.46 mg/g (Hexane Fr.), 0.79 mg/g (CHCl_3_ Fr.), 15.37 mg/g (EtOAc Fr.), 3.53 mg/g (BuOH Fr.), and 0.86 mg/g (H_2_O Fr.). Other compounds, such as erycibelline, 5-hydroxy-2-pyridinemethanol, m-hydroxyphenylglycine, *p*-aminophenol, and 4-hydroxycoumarin (data not shown) were present at lower concentrations in 5-*u*RCK, 5-*u*RCC, and these fractions. The ellagic acid content was not significantly different between each of the corresponding 5-*u*RCK and 5-*u*RCC fractions.

### 3.3. Effect of 5-uRCK Fractions and Ellagic Acid on Lipid Accumulation in FFA-Induced HepG2 Cells

**Figure 3:**
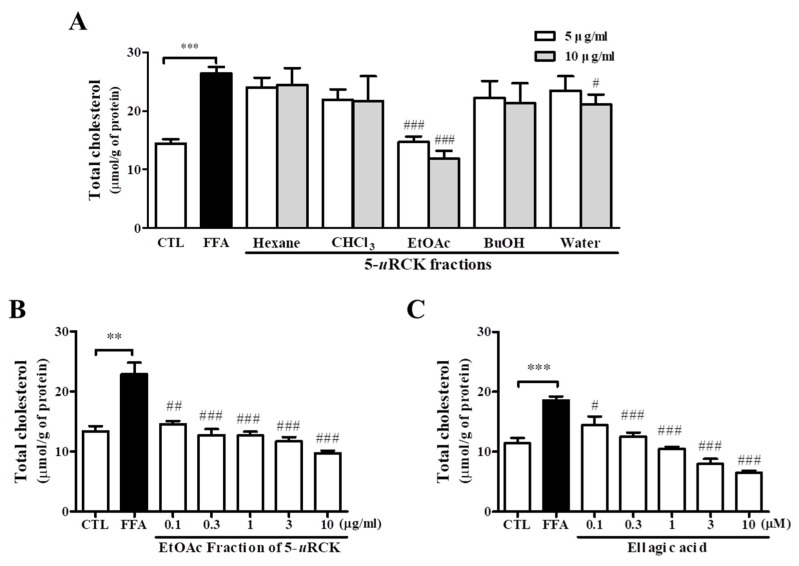
Effects of fractions of 5-*u*RCK or ellagic acid on cholesterol accumulation in FFA-induced HepG2 cells. HepG2 cells were treated with a 1 mM FFA mixture (oleic acid/palmitic acid, 2:1) and various concentrations of 5-*u*RCK fractions or ellagic acid for 24 h. (**A**) The effect of various fractions of 5-*u*RCK or 5-*u*RCC on the intracellular total cholesterol content in FFA-induced HepG2 cells. (**B**) The effect of the ethyl acetate fraction of 5-*u*RCK on the intracellular total cholesterol content in FFA-induced HepG2 cells. (**C**) The effect of purified ellagic acid from 5-*u*RCK on the intracellular total cholesterol content in FFA-induced HepG2 cells. ** *p* < 0.01, *** *p* < 0.001 versus control (CTL). ^#^ *p* < 0.05, ^##^ *p* < 0.01, ^###^ *p* < 0.001 versus FFA-treated cells. The data are represented as the means ± SEMs.

To determine if fractions of 5-*u*RCK and ellagic acid had an effect on the TC content in HepG2 cells, we treated them with five different fractions of 5-*u*RCK and ellagic acid, which is its main compound, at various concentrations for 24 h (Figure 3). The EtOAc fraction of 5-*u*RCK had the strongest TC-lowering effect and was even significantly stronger than that of the total 5-*u*RCK extract. To confirm the TC-lowering effect of the EtOAc fraction of 5-*u*RCK, we quantified the TC content. The TC content was dose-dependently decreased in HepG2 cells treated with the EtOAc fraction of 5-*u*RCK (Figure 3B). Furthermore, exposure to ellagic acid for 24 h potently and dose-dependently decreased TC levels in HepG2 cells (Figure 3C). A significant decrease by 22.42% was achieved by treatment with the EtOAc fraction of 5-*u*RCK at 100 nM, and a decrease by 60–70% was reached following treatment with the EtOAc fraction of 5-*u*RCK at 3–10 μM.

### 3.4. Effects of 5-uRCK and Ellagic Acid on the Growth Parameters and Organ Tissue Weights of Rats Fed an HCD

**Figure 4:**
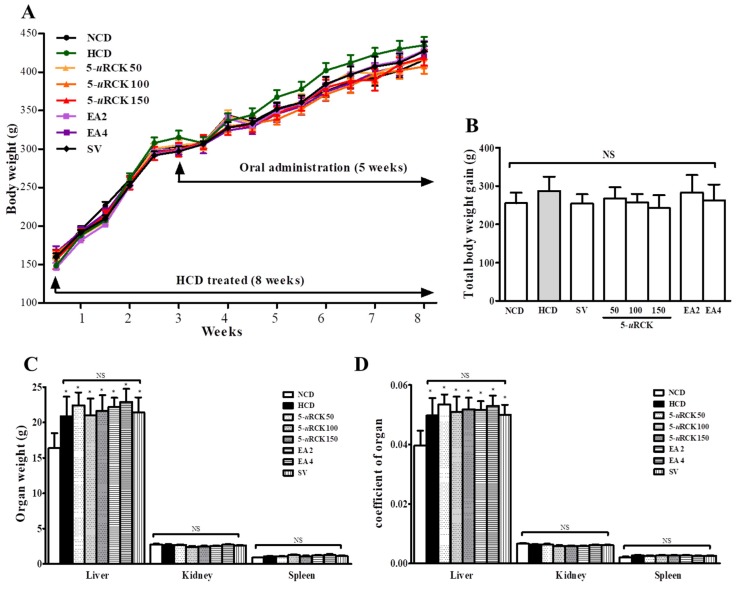
Effects of 5-*u*RCK and ellagic acid (EA) on body and organ weights in rats fed a high-cholesterol diet (HCD). (**A**) Body weight was measured every week for eight weeks of general (NCD) or HCD diet supplemented with or without 5-*u*RCK, ellagic acid, or simvastatin. (Figure S2). (**B**) Body weight gain in hypercholesterolemic rats fed an HCD for eight weeks. (**C**) Effect of 5-*u*RCK and ellagic acid on organ (liver, kidney, and spleen) weight in hypercholesterolemic rats fed an HCD. (**D**) Comparison of liver, kidney, and spleen weight ratio after 5-*u*RCK, ellagic acid, or simvastatin treatment for five weeks. The liver, kidney, and spleen weight ratio is equal to the liver, kidney, and spleen wet weight/body weight × 100%. *p* values compared with controls (NCD group) are denoted as * *p* < 0.05. The results are expressed as the mean ± SD (*n* = 8).

Figure 4A,B shows the total body weights of rats at the starting and final points of the experiments. Although the average total body weights of each group were almost the same at the starting point, the body weight gains in the 5-*u*RCK-treated and ellagic acid-treated groups were slightly lower than those in the HCD group in the final week (*p* > 0.05). However, the final liver weight and liver weight ratios (liver index) were significantly increased by 127.45% in the HCD group compared to the NCD group (*p* < 0.05), whereas liver weight and ratio were not significantly reduced following treatment with 5-*u*RCK and ellagic acid at all doses for five weeks (Figure 4C,D). In addition, liver weight increased significantly (*p* < 0.05) in all treatment groups compared to the NCD group. Additionally, there was no significant difference in body weight and liver weight between rats in the simvastatin group and the HCD group. There were no differences in the kidney and spleen weights. In case of the feed efficiency ratio of rats in all control and treated groups, no significant changes were observed (data not shown). In contrast, 5-*u*RCK and ellagic acid treatment protected against liver damage was caused by chronic HCD intake by lowering the elevated ALT and AST levels (Table 4). The levels of ALT and AST, which are key biomarkers of liver damage, were analyzed. There was a significant increase in the serum AST and ALT levels in the HCD group, whereas 5-*u*RCK treatment significantly decreased serum AST and ALT levels. In addition, the ellagic acid groups had significantly decreased serum AST and ALT levels compared to those in the HCD group. The 5-*u*RCK, ellagic acid, and simvastatin groups exhibited similar effects. These results indicate that the oral administration of 5-*u*RCK and ellagic acid for five weeks reduced liver toxicity in HCD-fed rats as a hypercholesterolemia rat model and was safe.

### 3.5. 5-uRCK and Ellagic Acid Regulated Serum Lipids in HCD Diet-Induced Hypercholesterolemic Rats

As shown in Table 4, when compared with the control group, the HCD group showed markedly increased serum TC, TG, LDL/VLDL-C, and Apo B levels. Levels of the serum lipid parameters TC, TG, LDL/VLDL-C, and Apo B were decreased, whereas HDL-C and Apo A1 levels were increased in the 5-*u*RCK-treated and ellagic acid-treated groups compared with the HCD group. Rats fed an HCD supplemented with simvastatin had lower TC, TG, LDL/VLDL-C, and Apo B levels, while only TG, LDL/VLDL-C, and Apo B levels were significantly decreased compared to those in the HCD group. We further measured the serum HDL-C level and found that, similar to simvastatin, 5-*u*RCK and ellagic acid efficiently enhanced serum HDL-C levels in HCD-fed rats. The non-HDL-C levels were calculated by subtracting the HDL-C levels from the TC levels. 5-*u*RCK at 100 mg/kg and 150 mg/kg and ellagic acid at 4 mg/kg significantly reduced serum non-HDL-C levels by 38.65% (*p* < 0.01), 53.41% (*p* < 0.001), and 45.49% (*p* < 0.01), respectively, when compared with those in the HCD group (data not shown). AI_1_ and the CRI atherogenic index indicate the risk of atherosclerosis. A lower value indicates a lower risk of atherosclerosis. The AI_1_ and CRI values were calculated and found to be significantly elevated in all groups that received the HCD compared to those that received the NCD. The highest increase in AI_1_ and CRI among the treatment groups was observed in the group that received only an HCD. The differences in AI_1_ and CRI between the HCD and control groups were statistically significant (*p* < 0.001). The AI_1_ and CRI in the group that received a HCD, 5-*u*RCK, and ellagic acid were also elevated compared to those in the NCD group. However, the 5-*u*RCK group and the ellagic acid group were significantly lower than those in the group treated with only HCD.

### 3.6. Effects of 5-uRCK and Ellagic Acid on the Hepatic Lipid Levels of HCD-Fed Rats

As shown in Table 5, liver tissue from the HCD group had significantly increased TC, FC, and CE levels compared to the levels from the NCD group. Administration of 5-*u*RCK significantly decreased the increased levels of TC, FC, and CE in HCD-fed rats. In addition, treatment with ellagic acid significantly reduced the elevated levels of TC, FC, and CE in the livers of HCD-fed rats at week 8. Similarly, treatment with simvastatin significantly reduced the elevated levels of TC, FC, and CE in the livers of HCD-fed rats, even though its inhibitory activity was lower than that of ellagic acid (4 mg/kg). Moreover, the ratio of free cholesterol to cholesteryl ester was also significantly increased in 5-*u*RCK-treated cells (0.57 vs. 0.92 for the HCD group and 5-*u*RCK 150 group, respectively). In particular, the 5-*u*RCK 150 group had levels similar to those of the ellagic acid and simvastatin groups.

### 3.7. 5-uRCK and Ellagic Acid Regulated Hepatic Antioxidant Enzyme Activities in HCD-Fed Rats

As shown in Table 6, the antioxidant parameters of the liver (TAOC, SOD, GSH-Px, and CAT) in the HCD group were decreased, and the oxidative damage parameters (MDA and protein carbonyl content) were concomitantly increased. Compared with the HCD group, the 5-*u*RCK and ellagic acid treatment groups showed significant antioxidant effects in a dose-dependent manner, except for the group fed 5-*u*RCK at 50 mg/kg. In particular, the TAOC and SOD and GSH-Px levels were restored by 5-*u*RCK (150 mg/kg) and ellagic acid (4 mg/kg) administration. Conversely, liver MDA and protein carbonyl levels were upregulated in the HCD group when compared with the NCD group. The administration of 5-*u*RCK at 100–150 mg/kg and ellagic acid at 2–4 mg/kg to HCD-fed rats significantly decreased the MDA levels. The administration of 5-*u*RCK at 150 mg/kg and ellagic acid at 4 mg/kg with dietary HCD had a significant effect on the protein carbonyl content.

### 3.8. Effects of 5-uRCK and Ellagic Acid on Lipid Accumulation in the Liver Tissues of HCD-Fed Rats

**Figure 5:**
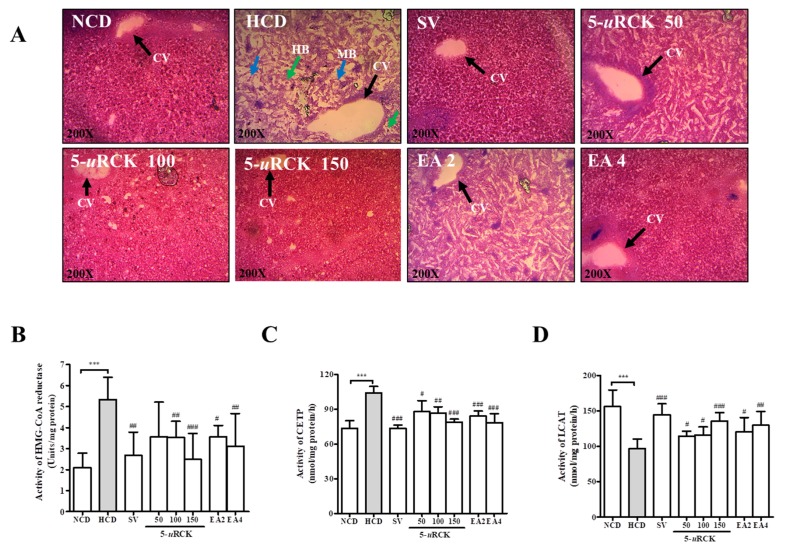
5-*u*RCK and ellagic acid supplementation improved liver steatosis in hypercholesterolemic rats fed a high-cholesterol diet (HCD) for eight weeks. (**A**) Liver histological changes were observed after five weeks of 5-*u*RCK, ellagic acid, or simvastatin treatment. Liver sections stained with H&E (magnification, 200×). (**B**) In vivo modulatory effect of 5-*u*RCK and ellagic acid on hepatic HMG-CoA reductase activity in HCD-fed rats. (**C**) In vivo modulatory effect of 5-*u*RCK and ellagic acid on hepatic cholesteryl ester transfer protein (CETP) activity in high-cholesterol diet (HCD)-fed rats. (**D**) An in vivo modulatory effect of 5-*u*RCK and ellagic acid on hepatic lecithin cholesterol acyltransferase (LCAT) activity in HCD-fed rats. The arrow indicates the central vein (CV), hepatocyte ballooning (HB), and Mallory-Denk body (MB). *** *p* < 0.001 versus control (NCD) group, ^#^ *p* < 0.05, ^##^ *p* < 0.01, ^###^ *p* < 0.001 versus the HCD group. All data are shown as the mean ± SD (*n* = 6–8).

To identify the effects of 5-*u*RCK and ellagic acid on HCD-induced lipid accumulation in experimental animals, tissue samples were prepared from the livers of rats in the treatment and in NCD groups. The samples were subjected to H&E staining (Figure 5A). In the HCD group, hepatocyte ballooning with Mallory-Denk bodies and lipid droplets were clearly observed. These histological abnormalities were reduced in the liver tissues of rats treated with 5-*u*RCK and ellagic acid in a dose-dependent fashion.

### 3.9. Effects of 5-uRCK and Ellagic Acid on Hepatic HMG-CoA Reductase, CETP, and LCAT Activities in HCD-Fed Rats

As shown in Figure 5B, hepatic HMG-CoA reductase activity was significantly increased in the HCD group (*p* < 0.001). The results showed that 5-*u*RCK and ellagic acid inhibited enzyme activity in a concentration-dependent manner. 5-*u*RCK at 100 and 150 mg/kg inhibited the HMG-CoA reductase enzyme activity by 33.40% (*p* < 0.01) and 53.28% (*p* < 0.001), respectively. Ellagic acid at 4 mg/kg significantly inhibited enzyme activity by 41.46% (*p* < 0.01).

As shown in Figure 5C, higher CETP activities were found, and CETP was significantly increased in the HCD group (*p* < 0.001). The 5-*u*RCK, ellagic acid, and simvastatin groups showed the effective downregulation of CETP levels in a dose-dependent manner compared with those in the HCD group.

As shown in Figure 5D, hepatic LCAT activity was markedly decreased in the HCD group compared to the NCD group. Administration of 5-*u*RCK and ellagic acid significantly increased LCAT activity when compared with the administration in the HCD group. Furthermore, the LCAT activity was significantly increased in the simvastatin group compared with the 5-*u*RCK and ellagic acid groups.

### 3.10. 5-uRCK and Ellagic Acid Ameliorated Atherosclerosis in HCD-Fed SD Rats

**Figure 6:**
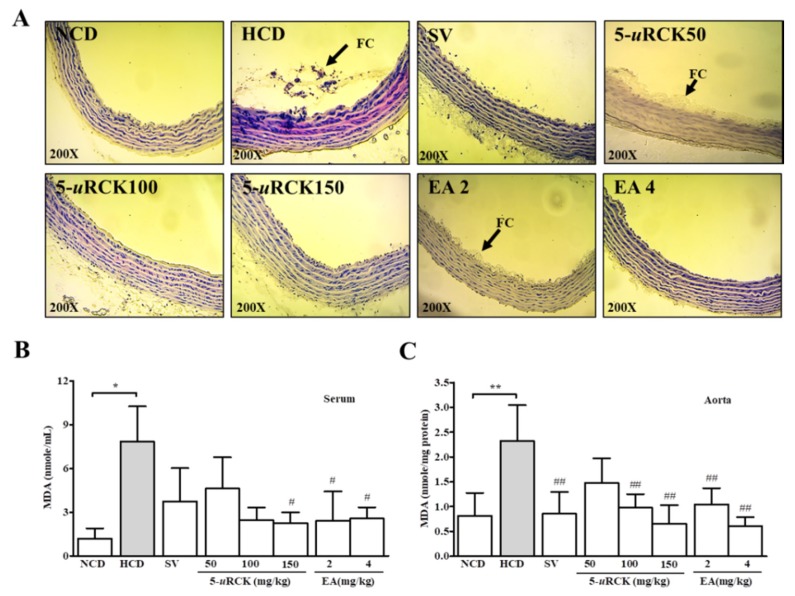
Photomicrographs of H and E-stained abdominal rat aortas after different treatments for 5 weeks (**A**). Effect of dietary supplementation with 5-*u*RCK or ellagic acid on serum (**B**) and aortic (**C**) malondialdehyde (MDA) levels in HCD-fed rats. The arrows point to foam cells (FC). * *p* < 0.05, ** *p* < 0.01, versus the control (NCD) group, ^#^ *p* < 0.05, ^##^ *p* < 0.01, ^###^ *p* < 0.001 versus the HCD group. All data are shown as the mean ± SD (*n* = 6–8).

Sections of aorta samples were stained by H and E, as shown in Figure 6A. There were no pathological changes in the aortas of rats in the NCD group, but rats that consumed an HCD showed a thickening of atheromatous plaque formations and the presence of foam cells. The pathological changes in the aortas of rats in the 5-*u*RCK (50 mg/kg)-treated group and the ellagic acid (2 mg/kg)-treated group were less visible than those in the HCD group, while photomicrographic examination of the aortas of rats from the groups treated with 5-*u*RCK (100 mg/kg), 5-*u*RCK (150 mg/kg), and ellagic acid (4 mg/kg) showed normal intimal medial thickening compared to that in the NCD group.

### 3.11. Effects of 5-uRCK and Ellagic Acid on Serum and Aortic Lipid Peroxidation Activity in HCD-Fed SD Rats

Regarding the serum and aortic TBARS (MDA) levels, an increase in lipid peroxidation was observed in the HCD group compared with the normal group (Figure 6B,C). The group administered 5-*u*RCK at 150 mg/kg had significantly reduced serum TBARS levels compared with those in the HCD group. However, the groups administered 5-*u*RCK at 50 mg/kg and 100 mg/kg did not have serum TBARS levels significantly different than those in the HCD group. The groups administered ellagic acid at 2 mg/kg and 4 mg/kg had notably lower TBARS levels compared with those in the HCD group. The groups administered 5-*u*RCK at 100 mg/kg and 150 mg/kg had significantly reduced aortic TBARS levels compared with those in the HCD group. Additionally, the group treated with ellagic acid at 2 mg/kg and 4 mg/kg had notably lower TBARS levels than the HCD group.

### 3.12. 5-uRCK and Ellagic Acid Decreased Cholesterol Synthesis by Suppressing the Expression of SREBP-2 and HMGCR in the Liver

**Figure 7:**
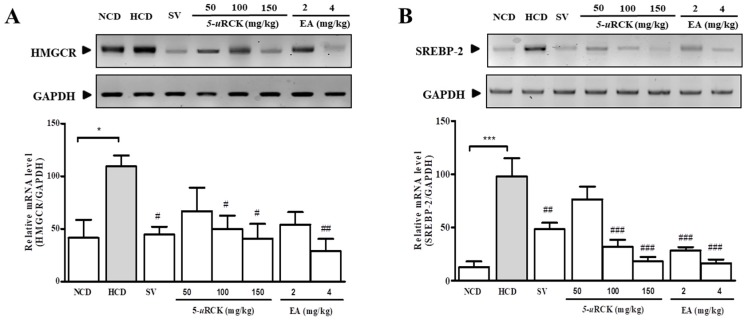
Effects of 5-*u*RCK or ellagic acid (EA) on the expression of HMGCR and SREBP-2 in the livers of rats fed an HCD. Relative mRNA expression of 3-hydroxy-3-methyl-glutaryl-CoA reductase (HMGCR) (**A**) and SREBP-2 (**B**) was analyzed after 5-*u*RCK or ellagic acid treatment for five weeks. The gene expression levels were quantified by qPCR. Experiment was repeated independently three times (*n* = 6–8 rats per group each time) and the RT-PCR product is representative of one of them. Each bar graph represents the mean ± SD of 6–8 rats per group (*n* = 6–8 rats/group, 3 times repeat). *P* values for comparisons with control groups (NCD groups) are denoted as * *p* < 0.05 and *** *p* < 0.001, and those for comparisons with HCD groups are denoted as ^#^ *p* < 0.05, ^##^ *p* < 0.01, and ^###^ *p* < 0.001.

The mRNA levels of SREBP-2 (*p* < 0.001) and HMGCR (*p* < 0.05) were increased in the HCD group when compared to the NCD group (Figure 7A,B). 5-*u*RCK and ellagic acid downregulated the gene expression of SREBP-2 and transcription levels of its target HMGCR in a dose-dependent manner.

### 3.13. Effects of 5-uRCK and Ellagic Acid on AMPK Phosphorylation and Lipid Metabolism in HCD-Fed Rats

**Figure 8:**
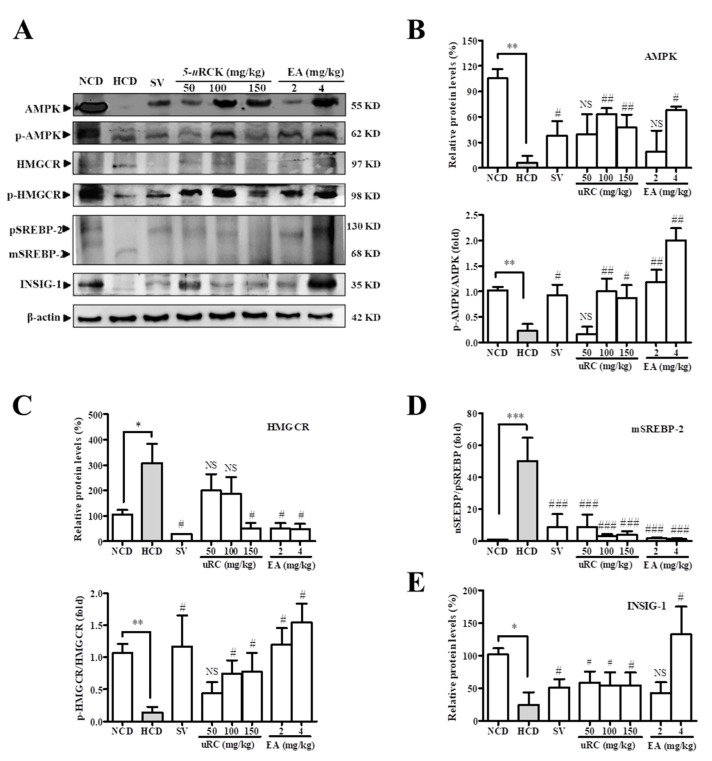
Effects of 5-*u*RCK or ellagic acid (EA) on the phosphorylation of AMPK and the expression of cholesterol biosynthesis-related proteins in the livers of rats fed an HCD. (**A**) The protein expression levels of p-AMPK, AMPK, p-HMGCR, HMGCR, precursor SREBP-2 (pSREBP-2), mature SREBP-2 (mSREBP-2), and INSIG-1 in liver tissues were measured by Western blotting. (**B**) The bar graphs indicate the average p-AMPK and AMPK levels. (**C)** The bar graphs indicate the average p-HMGCR and HMGCR levels. (**D**) The bar graph indicates the average pSREBP-2 and mSREBP-2 levels. (**E**) The bar graph indicates the average INSIG-1 levels. The data are expressed as the mean ± SD. *P* values for comparisons with control groups (NCD groups) are denoted as * *p* < 0.05, ** *p* < 0.01 and *** *p* < 0.001, and those for comparisons with HCD groups are denoted as ^#^ *p* < 0.05, ^##^ *p* < 0.01, and ^###^ *p* < 0.001.

As shown in Figure 8, the HCD group had significantly decreased expression of AMPK, phosphorylated AMPK (p-AMPK), phosphorylated HMGCR (p-HMGCR), and INSIG-1. Consistent with the RT-PCR results, the protein levels of HMGCR and mature SREBP-2 (mSREBP-2) were significantly increased in the HCD group when compared to the NCD group. Compared to the HCD group, the 5-*u*RCK and ellagic acid treatment groups had significantly increased expression of AMPK, p-AMPK, p-HMGCR, and INSIG-1 in a dose-dependent manner and decreased expression of HMGCR and mSREBP-2. The 5-*u*RCK and ellagic acid groups showed protein expression patterns similar to those in the simvastatin group. Collectively, these results suggest that 5-*u*RCK and ellagic acid directly activate AMPK, which may reduce intracellular cholesterol accumulation through the phosphorylation at HMGCR in vivo.

## 4. Discussion

The pharmacological activity of natural compounds has been proven scientifically and applied for the prevention of chronic diseases including inflammation and CVD [31]. In the present study, 5-*u*RCK was shown to contain a high concentration of ellagic acid. 5-*u*RCK displayed hypocholesterolemic activity and ameliorated hepatic steatosis. We identified natural materials as candidates to treat hypercholesterolemia by in vitro screening assays to detect lipid accumulation in 3T3-L1 adipocytes and intracellular TC content in HepG2 cells. We previously reported that a 5% ethanol extract of *u*RCK (5-*u*RCK, standardized ethanol extract) had anti-obesity activity in high-fat diet (HFD)-fed mice [22,23] and hypocholesterolemic effects in HCD-fed rats [24]. In this study, we confirmed that 5-*u*RCK and 5-*u*RCC attenuate hepatic fat accumulation in a dose-dependent manner by measuring the hepatic TC in FFA-induced hepatic steatosis using HepG2 cells (Figure 1). We also tested the inhibitory effect of 5-*u*RCK and 5-*u*RCC fractions on the induction of fat overload in HepG2 cells. In the current study, only EtOAc fractions of 5-*u*RCK or 5-*u*RCC were found to have a high content of ellagic acid (Figure 2). Furthermore, the EtOAc fractions (up to 10 μg/mL) of 5-*u*RCK and 5-*u*RCC and ellagic acid had the most substantial effect on downregulating TC levels in HepG2 cells (Figure 3) in a dose-dependent manner. We previously published the results of 5-*u*RCK compounds (ellagic acid, erycibelline, 5-hydroxy-2-pyridinemethanol, *m*-hydroxyphenylglycine, *p*-aminophenol, and 4-hydroxycoumarin) analysis [23,25]. Among them, ellagic acid decreased lipid accumulation and expression levels of the key adipogenic genes peroxisome proliferator-activated receptor γ (PPARγ), CCATT/enhancer-binding protein α (C/EBPα), sterol regulatory element binding protein-1c (SREBP-1c), acetyl-CoA carboxylase (ACC), and fatty acid synthase (FAS) to the greatest extent.

Based on these results, we investigated the hypocholesterolemic effects of 5-*u*RCK and ellagic acid in an HCD-induced hypercholesterolemia rat model. Body weight gain and food intake did not differ significantly among groups during the experimental period (Figure 4). The relative liver weights (per 100 g of body weight) were not significantly decreased in the 5-*u*RCK and ellagic acid groups compared to the HCD group. Moreover, the relative weights of the spleen and kidney were not significantly different among the groups. In our previous study, the oral administration of 5-*u*RCK at 50 mg/kg and 300 mg/kg did not decrease body weight gain in HCD-fed rats [24]. In this study, 5-*u*RCK and ellagic acid were administered at 50 mg/kg, 150 mg/kg, and 2 mg/kg per 4 mg/kg, respectively, which are the same doses previously used.

As for the liver and serum biochemical parameters, 5-*u*RCK and ellagic acid effectively reduced the levels of TG, TC, and LDL-C compared with those in the HCD group (Table 4 and Table 5) in addition to the level of TC in the liver plasma (Table 4). In contrast, 5-*u*RCK and ellagic acid significantly increased the serum levels of HDL-C. 5-*u*RCK and ellagic acid prevent liver damage, as indicated by reduced levels of liver damage marker ALT and AST, which were increased by HCD treatment (Table 4). Both AI and CRI were significantly reduced in all experimental diet groups compared with the HCD control group (Table 4). Most cholesterol is an essential structural element of the biological membrane, and the rest transits through the blood or functions as the starting material for the synthesis of bile acid, steroid hormones, and vitamin D. However, increased serum concentrations TC and LCL-C raise the risk of CVD [32]. Therefore, our results suggest that 5-*u*RCK and ellagic acid are beneficial for preventing diseases associated with arteriosclerosis and cardiac failure by improving lipid metabolism. The liver lipid profile exhibited a tendency similar to that of the serum lipid profile. As shown in Table 5, a cholesterol-enriched diet promoted the accumulation of liver lipids, and TC, FC, and CE content in the HCD group was increased by 3.42-fold, 2.58-fold, and 8.85-fold, respectively, than that in the NCD group (*p* < 0.001). However, the consumption of 5-*u*RCK and ellagic acid reduced lipid levels. Temel and others [33] reported that reduced hepatic CE content inhibits cholesterol absorption. Decreased hepatic FC available after acyl coenzyme A-cholesterol acyltransferase (ACAT) inhibition was very rapidly directed for its elimination in the bile directly or after conversion to bile acids [34]. In the present study, decreased hepatic FC and CE storage in the 5-*u*RCK and ellagic acid groups might be the main factor responsible for the effective inhibition of cholesterol absorption.

In our present study, the enzyme activity of HMG-CoA reductase was significantly downregulated compared to that in the HCD group by treating with only 5-*u*RCK (100–150 mg/kg) and ellagic acid (2–4 mg/kg) (Figure 5B). These results implied that 5-*u*RCK and ellagic acid prevent hypercholesterolemia and the formation of fatty liver by regulating the activities of enzymes that are important in cholesterol metabolism. CETP, which transfers cholesteryl ester from HDL-C and Apo B-containing lipoprotein, plays an important role in regulating the concentration and composition of HDL-C. A CETP inhibitor would be expected to increase plasma HDL-C levels and decrease LDL-C levels and, thus, serve as a potential therapeutic benefit for coronary artery disease. As shown in Figure 5C, treatment with 5-*u*RCK or ellagic acid was more effective in downregulating the CETP level in a dose-dependent manner. However, CETP inhibition may be potentially harmful, as there are studies showing that the incidence of cardiovascular disease was inversely related to plasma CETP, and certain alleles of the CETP gene that lower hepatic CETP secretion have been found to be associated with an increased risk of myocardial infarction [35,36]. Therefore, further extensive studies are needed to develop new cardiovascular drugs and to improve understanding of regulatory mechanisms with improved therapeutic profiles and reduced side-effects. The LCAT enzyme is bound to HDL-C and reacts preferentially with HDL-C [37]. LCAT deficiency is associated with a significant reduction in plasma HDL-C concentration [38]. As shown in Figure 5D, treatment with 5-*u*RCK or ellagic acid significantly increased hepatic LCAT activity in a dose-dependent manner.

A higher TBARS content was observed in the HCD group when compared with the NCD group, which indicates that oxidative stress was increased by an HCD. Administration of 5-*u*RCK and ellagic acid resulted in a decrease in the TBARS content in the liver (Table 6), serum (Figure 6B), and aorta (Figure 6C), which indicates that the lipid peroxidation induced by oxidative stress was inhibited. MDA, which is the product of lipid peroxidation, is an index of the level of oxygen free radicals. The obtained data suggested that 5-*u*RCK and ellagic acid have a reducing cholesterol accumulation and are therapeutic options in treating hypercholesterolemia.

A study has shown that hypercholesterolemia diminishes the antioxidant defense system and decreases the activities of SOD and CAT, which elevates the lipid peroxide content [39]. In the present study, the activities of TAOC, SOD, GSH-Px, and CAT in the liver of rats in the HCD group were significantly decreased when compared with those of rats in the NCD group. Administration of 5-*u*RCK and ellagic acid to rats fed a cholesterol-rich diet significantly elevated the activities of TAOC, SOD, GSH-Px, and CAT in the liver (Table 6). These results suggest that 5-*u*RCK and ellagic acid can improve the efficiency of the conversion of superoxide anion to hydrogen peroxide due to increased SOD activity, which catalyzes the dismutation of the superoxide anion into hydrogen peroxide. 5-*u*RCK and ellagic acid also increased the activity of CAT, which detoxifies hydrogen peroxide and converts lipid hydroperoxides to nontoxic substances in the liver. GSH-Px could transform toxic peroxide into a nontoxic hydroxyl compound and promote the decomposition of hydrogen peroxide to protect the structure and function of cell membranes from the damage and interference of peroxide. It is crucial to maintain enzyme activities to accommodate this oxidative stress and reduce oxidative damage [40]. In addition, combined with the results of the antioxidant experiments, these results clearly indicate that serum TC, TG, and AI were negatively correlated with antioxidant indicators (TAOC, SOD, GSH-Px, and CAT) and positively correlated with the MDA and protein carbonyl content. Lipid levels in the hyperlipidemic rats and their oxidation-antioxidant levels were shown to be closely related and mutually promotive. 5-*u*RCK and ellagic acid showed similar lipid-lowering and antioxidative effects and had significant physiological lipid-lowering and antioxidative activities.

The results from this study demonstrated that 5-*u*RCK significantly reduced the cholesterol concentration in vitro and in vivo by multiple mechanisms (Figure 7 and Figure 8). First, 5-*u*RCK suppresses HMGCR activity by direct phosphorylation. HMGCR, which is the rate-limiting enzyme in cholesterol biosynthesis, catalyzes mevalonate production from HMG-CoA. The phosphorylation of AMPK at Thr 172 was induced by 5-*u*RCK treatment, and active AMPK subsequently phosphorylated HMGCR at Ser 872 in rat livers, which indicates that 5-*u*RCK reduced the HMGCR enzyme activity in vivo. Second, 5-*u*RCK represses SREBP-2-dependent HMGCR transcription. In the post-translational modification of SREBP-2, AMPK-dependent phosphorylation of SREBP-2 hinders the ER-to-Golgi transport of pSREBP-2 [12]. The activation of AMPK may inhibit HMGCR gene expression by processing the phosphorylation inhibition of SREBP-2, which represses HMGCR transcription. Statins are known as a highly selective HMGCR inhibitor and the most effective side effects are known as muscle-related symptoms [5,6]. Although ellagic acid and 5-*u*RCK have the effect of inhibiting HMGCR, other complex mechanisms, especially AMPK-dependent mechanisms, are preferred. As a treatment for hypercholesterolemia, however, further studies are needed to investigate the long-term effects and safety of 5-*u*RCK that have HMGCR inhibitory effects such as statin. Third, we propose that INSIG-1, which is a key factor in SREBP-mediated regulation, decreases SREBP nuclear translocation. AMPK positively regulates INSIG-1 expression, and the transfection of AMPKα subunits induces INSIG-1 expression, whereas the transfection of dominant-negative AMPK suppresses the transcription of INSIG-1 [13]. Further studies are required to identify the effects of AMPK on INSIG-1 mRNA and protein expression. In this case, our data showed that 5-*u*RCK induced INSIG-1 expression and decreased levels of the active nuclear form of SREBP-2 with significance, which suggests that 5-*u*RCK substantially affects the activation of SREBP-2 in an AMPK-dependent manner. The potential anti-inflammatory role of 5-*u*RCK awaits further investigation. In vivo and in vitro studies have limited application to humans. Therefore, future clinical trials will need to demonstrate safety and efficacy. Further experiments are required. However, these results collectively indicate the activation of AMPK by 5-uRCK and ellagic acid achieved via multiple mechanisms.

## 5. Conclusions

In conclusion, our results suggested that 5-*u*RCK and ellagic acid had hypolipidemic effects in an HCD-induced hypercholesterolemic rat model. Our in vitro and in vivo findings provide evidence that 5-*u*RCK and ellagic acid provide advanced protection against HCD-related lipid accumulation and liver dysfunction and may be more effective functional treatments for managing hypercholesterolemia.

## Figures and Tables

**Table 1:** Extraction yields of 5-*u*RCK and 5-*u*RCC under 5% (*v*/*v*) aqueous ethanol-extracted conditions.

Taxon	Common Name	Type	Part Used	Extraction Condition	Extraction Yields [Dry Matter (%)]
*Rubus coreanus* Miquel	Korean blackberry (Bokbunja)	Korea cultivated	unripe fruit	20 volumes of 5% ethanol, 100 °C, 4 h	20.44 ± 0.61%
*Rubus chingii* Hu	Chinese raspberry (Bokbunja)	China cultivated	unripe fruit	20 volumes of 5% ethanol, 100 °C, 4 h	19.54 ± 0.73%

**Table 2:** Composition of the experimental diets fed to rats. The nutrition components are expressed as g/1000 g.

Ingredients	AIN-93G	Paigen’s Atherogenic Rodent Diet
Casein, 30 mesh	200.00	75.00
L-Cystein	3.00	0
Soy protein	0	130.00
DL-Methionine	0	2.00
Corn starch	397.486	275.00
Maltodextrin 10	132.00	150.00
Sucrose	100.00	30.00
Cellulose, BW200	50.00	90.00
Soy bean oil	70.00	50.00
*t*-Butylhydroquinone	0.014	0
Cocoa butter	0	75.00
Coconut oil 76	0	35.00
Mineral mix S10001	35.00	35.00
Calcium carbonate	0	5.50
Sodium chloride	0	8.00
Potassium citrate	0	10.00
Vitamin mix V10001	10.00	10.00
Choline bitartrate	2.50	2.00
Cholesterol, USP	0	12.50
Sodium cholic acid	0	5.00
FD&C red dye #40	0	0.10
Total (unit: g)	1000	1000.1

**Table 3:** Experimental group design.

Group	Number	Diet Composition	Death
Normal-cholesterol diet (NCD)	*n* = 8	Normal-cholesterol diet (8 weeks) + Saline (5 weeks)	*n* = 1
High-cholesterol diet (HCD)	*n* = 8	High-cholesterol diet (8 weeks) + Saline (5 weeks)	-
5-*u*RCK 50	*n* = 8	High-cholesterol diet (8 weeks) + 5-*u*RCK 50 mg/kg B.W. (5 weeks)	-
5-*u*RCK 100	*n* = 8	High-cholesterol diet (8 weeks) + 5-*u*RCK 100 mg/kg B.W. (5 weeks)	-
5-*u*RCK 150	*n* = 8	High-cholesterol diet (8 weeks) + 5-*u*RCK 150 mg/kg B.W. (5 weeks)	-
Ellagic acid 2 (EA 2)	*n* = 8	High-cholesterol diet (8 weeks) + Ellagic acid 2 mg/kg B.W. (5 weeks)	*n* = 2
Ellagic acid 4 (EA 4)	*n* = 8	High-cholesterol diet (8 weeks) + Ellagic acid 4 mg/kg B.W. (5 weeks)	*n* = 1
Simvastatin (SV)	*n* = 8	High-cholesterol diet (8 weeks) + Simvastatin 4 mg/kg B.W. (5 weeks)	-

**Table 4:** Changes in the serum biochemistry profiles, atherogenic index (AI), and cardiac risk index (CRI) determined from the sera of 5-*u*RCK-treated, ellagic acid-treated, or simvastatin-treated high-cholesterol diet (HCD)-fed rats. ** *p* < 0.01, *** *p* < 0.001 versus control (NCD) group, ^#^ *p* < 0.05, ^##^ *p* < 0.01, and ^###^ *p* < 0.001 versus the HCD group. All data are shown as the mean ± SD (*n* = 6–8).

Group.	Plasma Levels (mg/dl)								AI1	CRI
	TC	HDL-C	LDL/VLDL	TG	ALT	AST	Apo B	Apo A1		
NCD	82.68 ± 9.36	29.02 ± 0.90	13.57 ± 2.54	31.8 ± 5.10	23.00 ± 2.00	65.50 ± 7.51	1.89 ± 0.58	3.55 ± 0.35	1.80 ± 0.21	2.80 ± 0.21
HCD	158.8 ± 8.39 ***	16.57 ± 0.57 ***	78.00 ± 4.03 ***	107.5 ± 7.51 ***	40.50 ± 3.43 ***	110.50 ± 12.87 ***	6.33 ± 0.67 **	1.38 ± 0.20 **	8.90 ± 0.76 ***	9.90 ± 0.76 ***
5-*u*RCK 50	109.5 ± 5.56 ^###^	24.39 ± 1.82 ^##^	56.50 ± 1.73 ^###^	48.60 ± 4.76 ^###^	28.25 ± 1.32 ^#^	63.20 ± 3.96 ^###^	3.71 ± 0.72 ^#^	2.99 ± 0.17 ^##^	3.59 ± 0.36 ^###^	4.58 ± 0.36 ^###^
5-*u*RCK 100	112.6 ± 4.38 ^##^	25.34 ± 2.26 ^##^	60.75 ± 2.49 ^###^	37.33 ± 4.67 ^###^	27.67 ± 3.18 ^#^	62.00 ± 5.03 ^###^	3.25 ± 0.90 ^#^	2.90 ± 0.23 ^##^	3.40 ± 0.30 ^###^	4.39 ± 0.30 ^###^
5-*u*RCK 150	93.05 ± 7.41 ^###^	26.78 ± 1.22 ^###^	50.87 ± 1.73 ^###^	55.33 ± 5.98 ^###^	26.20 ± 2.71 ^##^	50.50 ± 2.63 ^###^	3.17 ± 0.77 ^##^	3.0 ± 0.20 ^##^	2.36 ± 0.24 ^###^	3.36 ± 0.24 ^###^
EA 2	144.5 ± 13.61	24.65 ± 2.48	68.91 ± 2.51	47.00 ± 4.21^###^	27.33 ± 1.56##	61.83 ± 3.39 ^###^	2.56 ± 0.59 ^#^	2.38 ± 0.22 ^#^	5.39 ± 0.54 ^###^	6.39 ± 0.54 ^###^
EA 4	98.19 ± 8.79 ^###^	20.66 ± 2.23 ^#^	66.96 ± 2.50 ^#^	49.00 ± 5.49 ^###^	21.20 ± 1.07 ^###^	64.75 ± 7.19 ^###^	2.44 ± 0.61 ^##^	2.06 ± 0.18 ^#^	3.66 ± 0.50 ^###^	4.66 ± 0.50 ^###^
SV	132.2 ± 6.16	25.85 ± 1.30 ^##^	66.61 ± 1.36 ^#^	53.67 ± 5.57 ^###^	26.20 ± 2.92 ^##^	55.75 ± 6.59 ^###^	2.03 ± 0.66 ^##^	2.25 ± 0.25 ^##^	4.21 ± 0.23 ^###^	5.21 ± 0.23 ^###^

**Table 5:** Levels of total cholesterol, free cholesterol, and cholesterol ester in the livers of 5-*u*RCK-treated or ellagic acid-treated HCD-fed rats. The total cholesterol, free cholesterol, and cholesterol ester levels and free cholesterol to cholesterol ester ratio levels were detected after 5-*u*RCK, ellagic acid, or simvastatin treatment for five weeks. 5-*u*RCK and ellagic acid supplementation decreased hepatic lipid levels in rats fed an HCD. *** *p* < 0.001 versus control (NCD) group. ^#^ *p* < 0.05, ^##^ *p* < 0.01, ^###^ *p* < 0.001 versus HCD group. All data are shown as the mean ± SD (*n* = 6–8).

Group	Total Cholesterol	Free Cholesterol	Cholesterol Ester	Free Cholesterol /Cholesterol Ester
	(mg/dL)			
Normal Cholesterol Diet (NCD)	26.77 ± 5.67	17.79 ± 10.27	7.10 ± 0.40	3.73 ± 0.37
HCD	91.55 ± 6.69 ***	45.96 ± 11.65 ***	62.87 ± 13.10 ***	0.57 ± 0.24 ***
5-*u*RCK 50	83.87 ± 9.21 ^#^	35.19 ± 9.16 ^###^	53.86 ± 11.06 ^###^	0.80 ± 0.43
5-*u*RCK 100	74.39 ± 9.71 ^###^	26.47 ± 8.14 ^###^	41.29 ± 17.0 ^###^	0.89 ± 0.32
5-*u*RCK 150	77.38 ± 4.16 ^###^	31.73 ± 3.87 ^###^	45.61 ± 4.04 ^###^	0.92 ± 0.34 ^#^
EA 2	71.02 ± 8.12 ^###^	32.02 ± 10.78 ^###^	47.84 ± 5.26 ^###^	0.83 ± 0.14 ^##^
EA 4	63.03 ± 13.41 ^###^	28.53 ± 11.33 ^###^	36.82 ± 7.50 ^###^	1.16 ± 0.31 ^###^
SV	73.89 ± 10.74 ^###^	34.96 ± 4.67 ^###^	34.25 ± 9.64 ^###^	1.10 ± 0.39 ^#^

**Table 6:** Effect of 5-*u*RCK or ellagic acid on hepatic antioxidant enzyme activities in HCD-fed rats. ** *p* < 0.01, *** *p* < 0.001 versus the control (NCD) group. ^#^ *p* < 0.05, ^##^ *p* < 0.01, ^###^ *p* < 0.001 versus the HCD group. All data was shown as the mean ± SD (*n* = 6–8).

Group	TAOC (U/mg Protein)	SOD (U/mg Protein)	GSH-Px (U/mg Protein)	CAT (U/mg Protein)	MDA (nmol/mg Protein)	Protein Carbonyl (nmol/mg Protein)
NCD	2.42 ± 0.15	132.6 ± 8.96	18.34 ± 3.40	73.10 ± 14.33	0.59 ± 0.50	0.81 ± 0.13
HCD	1.57 ± 0.23 ***	82.04 ± 3.96 ***	9.89 ± 1.89 **	27.20 ± 11.82 ***	2.96 ± 1.23 **	1.39 ± 0.11 ***
5-*u*RCK 50	1.90 ± 0.30	104.3 ± 35.79	10.35 ± 3.09	40.66 ± 15.25	1.62 ± 0.81	1.31 ± 0.06
5-*u*RCK 100	2.01 ± 0.27 ^#^	97.20 ± 38.97	17.28 ± 4.01 ^#^	52.52 ± 11.93 ^##^	0.94 ± 0.61 ^##^	1.23 ± 0.6
5-*u*RCK 150	2.23 ± 0.38 ^#^	143.2 ± 31.62 ^##^	17.74 ± 1.81 ^###^	59.00 ± 19.86 ^#^	0.95 ± 0.58 ^#^	0.94 ± 0.05 ^##^
EA 2	2.10 ± 0.24 ^##^	128.4 ± 29.27 ^#^	14.65 ± 4.12	53.64 ± 17.19^#^	0.68 ± 0.18 ^##^	1.11 ± 0.11
EA 4	2.45 ± 0.65 ^#^	118.8 ± 14.85 ^##^	16.42 ± 3.12 ^#^	52.37 ± 18.62 ^#^	0.65 ± 0.22 ^##^	1.0 ± 0.12 ^#^
SV	2.13 ± 0.22 ^##^	97.55 ± 15.96	17.58±4.43^#^	55.78 ± 14.90 ^#^	0.96 ± 0.41^##^	0.85 ± 0.07 ^###^

## Supplementary Materials

The following are available online at https://www.mdpi.com/2072-6643/12/3/610/s1. Figure S1. Cell viability of HepG2 cells following different concentrations of 5-uRCK or 5-uRCC exposure were measured by MTT assay; Figure S2. Effects of 5-uRCK and ellagic acid (EA) on body weights in rats fed a high-cholesterol diet (HCD).
