# Supplementary material for: Unripe Rubus coreanus Miquel Extract Containing Ellagic Acid Regulates AMPK, SREBP-2, HMGCR, and INSIG-1 Signaling and Cholesterol Metabolism In Vitro and In Vivo
Source: Nutrients. 2020 Feb 26;12(3):610. doi: 10.3390/nu12030610 (PMC7146129; doi:10.3390/nu12030610)
Supplement: Supplementary file 1 [file nutrients-12-00610-s001.pdf]

# 1. Supplementary method

**MTT assay for the measurement of cell viability.**

Cell viability was assessed by a MTT assay. HepG2 cells were seeded at a density of $1 \times 10^4$ cells/well in a 96-well plate and allowed to adhere overnight. Then, various concentrations of 5-*u*RCK or 5-*u*RCC were treated for 24 h. After addition of an MTT solution (5mg/ml; 50 μl/well) and incubation for 4 h, the supernatants were removed and the formazan crystals were solubilized in 100 μl DMSO. Optical density was determined at 540 nm. Cytotoxicity was expressed as a percentage relative to a control that contained no sample. Quantitative data were analyzed by using GraphPad Prism (GraphPad, Inc., San Diego, California, USA) software programs to obtain means and standard deviation (SD), n= number of independent experiments each with three replicates (n=2). Data were statistically evaluated using Student's t-test with GraphPad Prism software programs. Statistical significance was indicated when $p < 0.05$.

# 2. Supplementary Figure 1 and results

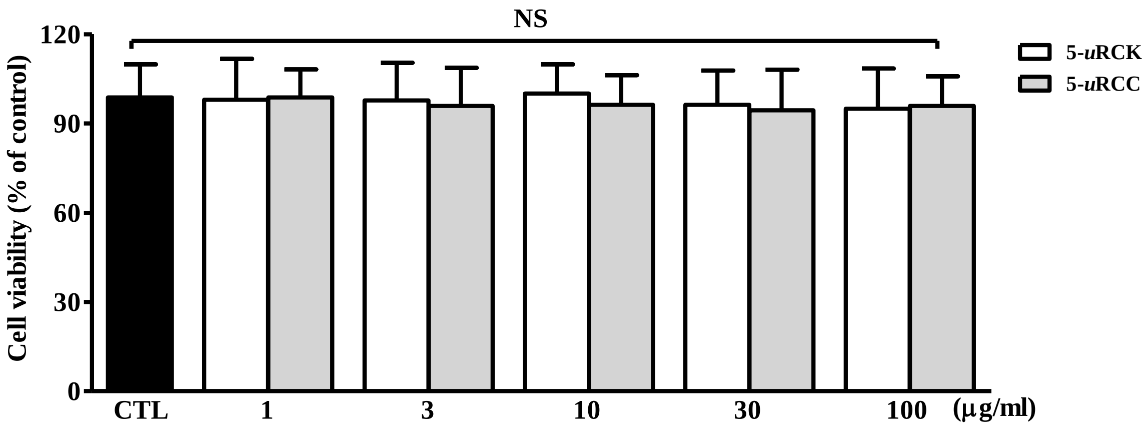


**Figure 1.** Cell viability of HepG2 cells following different concentrations of 5-*u*RCK or 5-*u*RCC exposure were measured by MTT assay (*n* = 2). HepG2 cells were exposed to different concentrations of 5-*u*RCK or 5-*u*RCC for 24 h. After exposure, cell viability for each treatment was determined based on spectrometry of formazan formation, and represented the viability percentage relative to control exposure. NS; not significant. The data are represented as the means ± SD.

The cytotoxicity of 5-*u*RCK was evaluated by MTT assay; the viability of cells treated with different concentrations (1, 3, 10, 30 and 100 μg/ml) of 5-*u*RCK compared to control cells was 98.76%, 98.74%, 95.88%, 96.29% and 94.91%, respectively. Furthermore, the results showed that the viability of the HepG2 cells in the presence of 5-*u*RCC at concentrations of 1, 3, 10, 30, and 100 μg/ml was not decreased (NS; $P > 0.05$). 5-*u*RCC led to a 97.96%, 97.74%, 96.27%, 94.44% and 95.88% viability compared to the control at concentrations of 1, 3, 10, 30 and 100 μg/ml, respectively. Therefore, it was decided that the noncytotoxic concentrations of up to 100 μM of the 5-*u*RCK and 5-*u*RCC would be used in the following experiments.

## 3. Supplementary Figure 2

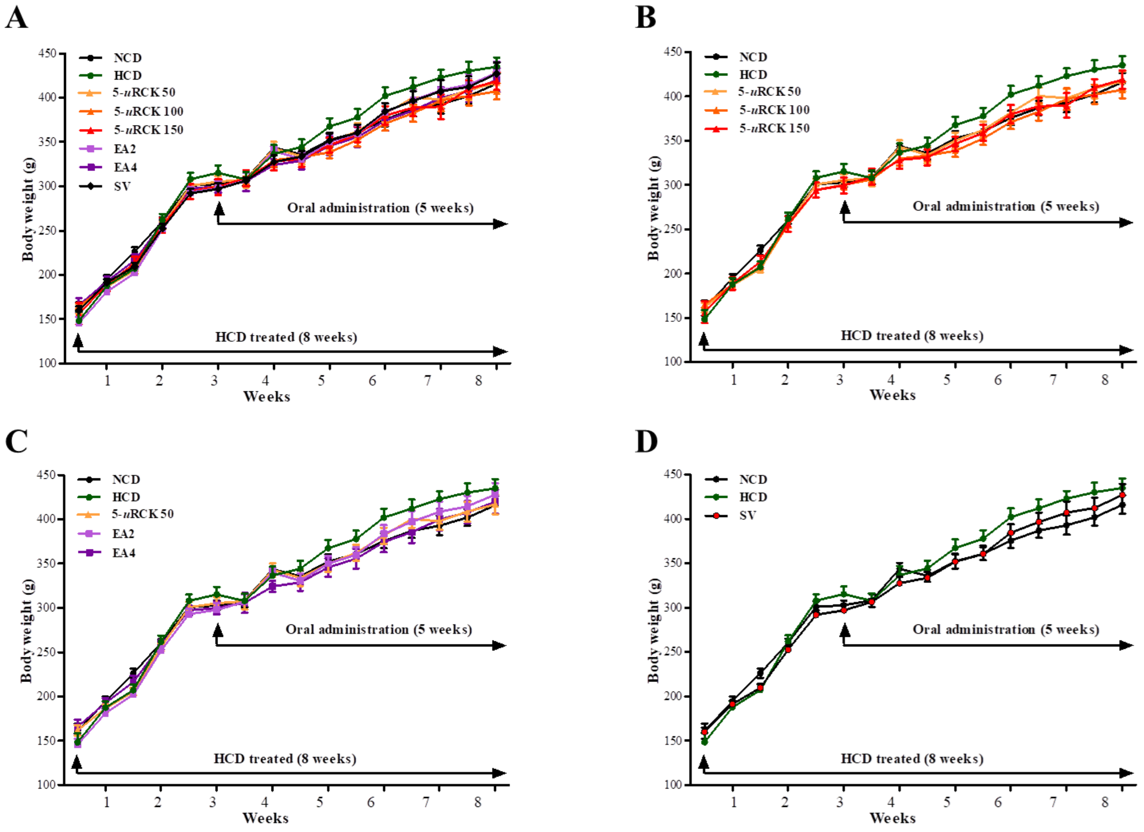


**Figure 2.** Effects of 5-*u*RCK and ellagic acid (EA) on body weights in rats fed a high-cholesterol diet (HCD). (**A**) Body weight was measured every week for eight weeks of general (NCD) or HCD diet supplemented with or without 5-uRCK, ellagic acid, or simvastatin. This result was separated to compare HCD vs (**B**) 5-uRCKs, (**C**) EAs and (**D**) simvastatin groups more clearly. The results are expressed as the mean ± SD. .
